# Macromolecular Crowding Enhances Catalytic Efficiency and Stability of *α*-Amylase

**DOI:** 10.5402/2013/737805

**Published:** 2012-11-08

**Authors:** Jay Kant Yadav

**Affiliations:** ^1^Department of Protein Chemistry and Technology, Central Food Technological Research Institute, Mysore 570 020, India; ^2^Max-Planck Research Unit for Enzymology of Protein Folding, Weinbergweg 22, 06120 Halle (Saale), Germany

## Abstract

In the present study an attempt was made to investigate the macromolecular crowding effect on functional attributes of *α*-amylase. High concentrations of sugar based cosolvents, (e.g., trehalose, sucrose, sorbitol, and glycerol) were used to mimic the macromolecular crowding environment (of cellular milieu) under *in vitro* conditions. To assess the effect of macromolecular crowding, the activity and structural properties of the enzyme were evaluated in the presence of different concentrations of the above cosolvents. Based on the results it is suggested that the macromolecular crowding significantly improves the catalytic efficiency of the enzyme with marginal change in the structure. Out of four cosolvents examined, trehalose was found to be the most effective in consistently enhancing thermal stability of the enzyme. Moreover, the relative effectiveness of the above cosolvents was found to be dependent on their concentration used.

## 1. Introduction

Proteins are complex molecules and often unstable when they are placed out of their native environment. Proteins or enzymes may also lose their activity as a result of high temperature, aggregation, proteolysis, and suboptimal solution conditions. The purified proteins are often unstable and need to be stabilized in order to maintain the structural integrity and activity. Protein aggregation during processing and formulation is one of the major setbacks that limit the rapid commercialization of protein-based pharmaceuticals. Proteins aggregation is usually triggered by the formation of partially unfolded intermediates and therefore the demand for successful stabilization protocol is progressively increasing [1]. The phenomenon of stabilization of proteins infers the preservation of the native protein structure and activity during storage. The protein stabilization is based on the principle of limiting the molecular motion and conformational transition while maintaining the protein still in the native state [2, 3]. The native structure of a protein is dictated by various intramolecular interactions and its contacts with the surrounding solutes and solvents. 

Several sugars and polyols have extensively been used to stabilize the proteins against denaturation and to extend their shelf life during storage [4, 5]. The structural stability of a protein in an aqueous solution is determined by several types of weak interactions. The interactions between solvent, cosolvents, and protein have often been explained in terms of transfer free energy, preferential interaction parameters and preferential interaction coefficients [6, 7]. Probably, the idea to stabilize the protein by using natural cosolvents must have come from the fact that many living organisms accumulate a number of osmolytes in response to adverse environmental stress conditions, which usually act as molecular crowders in the cytosol. Mounting evidences suggest that begins from archaebacteria to mammals, several mechanisms are evolved to protect the cells when they are subjected to hypertonic or stress conditions [8]. A recent report indicates that accumulation of compatible osmolytes provides a protective mechanism that can easily trigger the expression system specific for uptake of the osmolytes from the environments [9, 10]. It is likely that the behavior of an enzyme may be substantially different when it is sequestered in cytosol loaded with osmolytes and other macromolecular crowders, compared to the enzyme suspended in normal buffer. When an activity assay of an enzyme is performed in diluted buffer condition, it essentially does not correspond to large cytosolic macromolecular crowding environment and the observed activity may not be identical to its cytosolic counterpart. According to Zimmerman and Trach [11], the total protein concentration in an *E. coli* cell is use to be >300 mg/mL, which is equivalent to >30% (w/v). Such high concentration of macromolecules along with other available osmolytes may have critical effect on both conformation and catalytic activity of an enzyme. The mechanisms of cosolvent-induced effect on proteins are frequently explained in terms of preferential interactions and change in thermodynamic parameters, and the cosolvent-induced minor structural fine-tunings are often ignored. It is important to consider that the biological activities of enzymatic proteins are functions of minor conformational transitions. A protein under macromolecular crowding conditions may acquire a minor shift in the structure; nevertheless, such structural rearrangement could have an impact on its function. Considering the above perspectives, an attempt was made to investigate the effect of macromolecular crowding on activity and structure of a model enzyme. Addition of natural cosolvents (such as polyols and sugars) to the enzyme solution may imitate the conditions of macromolecular crowding to a certain extent that enable us to understand the possible effect on protein function. To explore this idea *α*-amylase (from *Bacillus amylolequifaciens*) was used as a model enzyme (as its structure is well known) in this investigation. The catalytic activity and thermal stability of the enzyme were examined in the presence of different concentrations of trehalose, sorbitol, sucrose, and glycerol.

*α*-Amylase (endo 1,4-*α*-D-glucan glucanohydrolases, EC: 3.2.1.1) randomly cleaves the *α*-D-1, 4-glycosidic bonds in starch with retention of *α*-anomeric configuration in the end product. It consists of three separate domains called domains A, B, and C and with specific (*α*/*β*)_8_ barrel, also known as TIM (triose phosphate isomerase) that forms the core of the enzyme molecule [12]. Domain B is formed by a protrusion between third *β*-strand and third helix of (*α*/*β*)_8_ barrel and it is rich in *β* structure [13]. The C domain constitutes C-terminal part of the sequence and forms a Greek key motif. The function of domain C is not fully known yet, but the mutation in this domain suggests that it has a vital role in enzymatic catalysis [14, 15]. The criteria for selection of these cosolvents were based on their solubility at higher concentration, biological compatibility and inertness towards any possible covalent interaction with protein molecules.

## 2. Materials and Methods

### 2.1. Materials

*α*-Amylase type II (from *Bacillus amylolequifaciens*), trehalose, sorbitol, sucrose, glycerol, starch, dinitrosalisylic acid, bovine serum albumin (BSA), sodium azide, and CaCl_2_ were procured from Sigma Chemicals Company, USA. Spectrapore dialysis membrane (MWCO, 6 KDa) was procured from Spectrum, Houston, USA. All chemicals were of analytical grade. The crystalline *α*-amylase was dissolved and dialyzed in 0.02 M citrate buffer, pH 5.9 to remove additives, freeze-dried (−80°C), and desiccated at −20°C for future use. Quartz triple distilled water was used for all the experiments.

### 2.2. Methods

#### 2.2.1. Treatment of the Enzyme with Cosolvents

Stock solutions of cosolvents and enzyme were prepared in 0.02 M sodium citrate buffer, pH 5.9. Then the enzyme was incubated in different concentration (ranging 0–40%, w/v) of each cosolvent (trehalose, sorbitol, sucrose, and glycerol) at 10°C for 48 hours with gentle agitation on a magnetic stirrer. Such treatment facilitates equilibration and preferential interaction of cosolvents with protein. After 48 hours of incubation, all the enzyme samples were centrifuged at 10000 rpm, at 4°C, for 20 min and supernatants were collected while leaving bottom 10% of its volume to avoid any precipitate. Protein concentration in each sample was determined by using Lowry method [16]. If required the enzyme samples were diluted to required concentration in the respective buffers or cosolvents solutions, before measuring activity, thermal stability, intrinsic fluorescence, and far UV-CD. All the solutions were supplemented with 0.005% (w/v) of sodium azide to avoid bacterial contamination and it did not show any inhibitory effect on the enzyme at this concentration.

#### 2.2.2. *α*-Amylase Activity Measurement

*α*-Amylase activity was measured by using method of Bernfeld [17] for estimation of reducing sugar. Reaction mixture, containing 1 mL of 1% starch solution and 1 mL of the enzyme solution, was incubated for 5 min at 37°C. The reaction was terminated by addition of 2 mL of 1% alkaline dinitrosalicylic acid solution. The whole solution was then subjected to heat in boiling water bath for 10 min. After cooling the solution was diluted five times using triple distilled water, mixed properly, and absorbance was recorded at 540 nm. Appropriate controls were set along with and subtracted from the values of enzyme samples. Each sample consisted of five replicates. The enzyme activity was calculated by using maltose standard. One unit of *α*-amylase was defined as amount of enzyme required to hydrolyze starch to produce 1 *μ*mol of reducing sugar (maltose) under given condition. 

#### 2.2.3. Assessment of Reversibility of Cosolvent-Induced Effect on Enzyme Activity

In order to assess the reversibility of cosolvent induced effect on *α*-amylase, the enzyme activity was measured before and after removal of cosolvents. The cosolvents from the enzyme solution were removed by using simple dialysis against buffer for 15 hrs at 10°C to eliminate all the cosolvents added with several changes of buffer at every two hours. At the end carbohydrate analysis of the enzyme samples was performed to ensure the complete removal of cosolvents from the enzyme sample. 

#### 2.2.4. Thermal Stability Measurement

The thermal inactivation of *α*-amylase was carried out in the presence of cosolvents trehalose, sorbitol, sucrose, and glycerol, using final concentration of the enzyme as 1 *μ*g/mL. The enzyme solutions were incubated at 60°C for 10 min and transferred onto ice and allowed to stand for 30 min before the enzyme activity was measured at 37°C. Assuming a simple two-step phenomenon for the irreversible thermal inactivation process, the half lives (*T*_1/2_) of the enzyme (at 60°C), in the presence of different cosolvents were calculated from the slope of the inactivation curves obtained by linear regression of residual activity versus incubation time [18]. The *T*_1/2_ represents an average of five independent values. *T*_1/2_ is defined as the time required to irreversibly reducing 50% of the enzyme activity under given condition.

#### 2.2.5. Intrinsic Fluorescence Measurement

Intrinsic fluorescence spectra of *α*-amylase (50 *μ*g/mL) were recorded at 25°C in the presence of different concentrations of cosolvents in a Shimadzu spectrofluorophotometer model RF-5000, equipped with temperature-controlled water bath. The fluorescence excitation wavelength was set at 280 nm and emission spectra were recorded in the range of 300–400 nm using slit width of 10 and 5 nm for excitation and emissions respectively. Each spectrum represents an average of three independent scans.

#### 2.2.6. Measurement of Circular Dichroism (CD)

The far UV-CD measurements of the enzyme in the presence of 30% (w/v) concentrations of cosolvents were carried out in the far-UV region (200–260 nm) on a Jasco spectropolarimeter model J-810 at 25°C. The instrument was calibrated with D-10 camphorsulphonic acid. The CD scan was performed by using 0.26 mg/mL of the enzyme with path length of 1 mm and 50 nm/min scan speed. The observed ellipticitiy was expressed as mean residue ellipticity (MRE) in deg-cm^2^ dmol^−1^. Each spectrum represents an average of five independent scans after subtracting the respective reference. 

## 3. Results 

### 3.1. Thermal Stability of *α*-Amylase in Cosolvents

**Figure 1:**
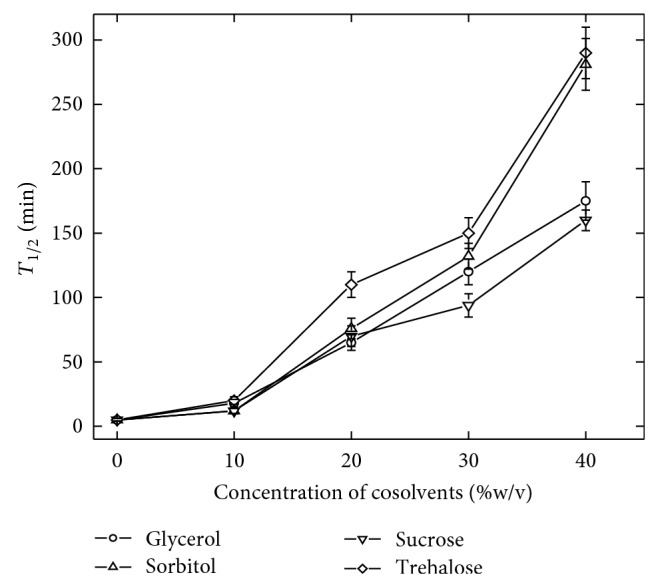
Effect of cosolvents on thermal stability of *α*-amylase. The enzyme solutions in the respective buffer or cosolvent solutions were incubated at 60°C for different period of time and residual activity was evaluated. The error bar represents the standard deviation.

Thermal stability of *α*-amylase was studied in the presence of four selected cosolvents, sorbitol, sucrose, trehalose, and glycerol. The rate of thermal inactivation of enzyme was significantly reduced in the presence of cosolvents. The stabilization effect of all the cosolvents was estimated by measuring *T*_1/2_ of the enzyme at 60°C. All the cosolvents were found to have significant effect on stability of the enzyme against temperature-induced inactivation. The effectiveness of each cosolvent was found to be linearly enhanced with increasing the concentration. The enzyme lost 50% of its activity after 5 min of incubation at 60°C. In the presence of various cosolvents the *T*_1/2_ value was found to be increased several folds and the increment was dependent on the concentration of cosolvents used (Figure 1). Although addition of all the four cosolvents was found to be useful in reducing the rate of thermal inactivation of the enzyme, trehalose was found to be most effective. At 40% concentration, trehalose displayed 58-fold enhancements in *T*_1/2_ of the enzyme compared to 32 fold, 56-fold, and 35-fold in the presence of sucrose, sorbitol, and glycerol (Figure 1), respectively. 

### 3.2. Cosolvent-Induced Enhancement in *α*-Amylase Activity

**Figure 2:**
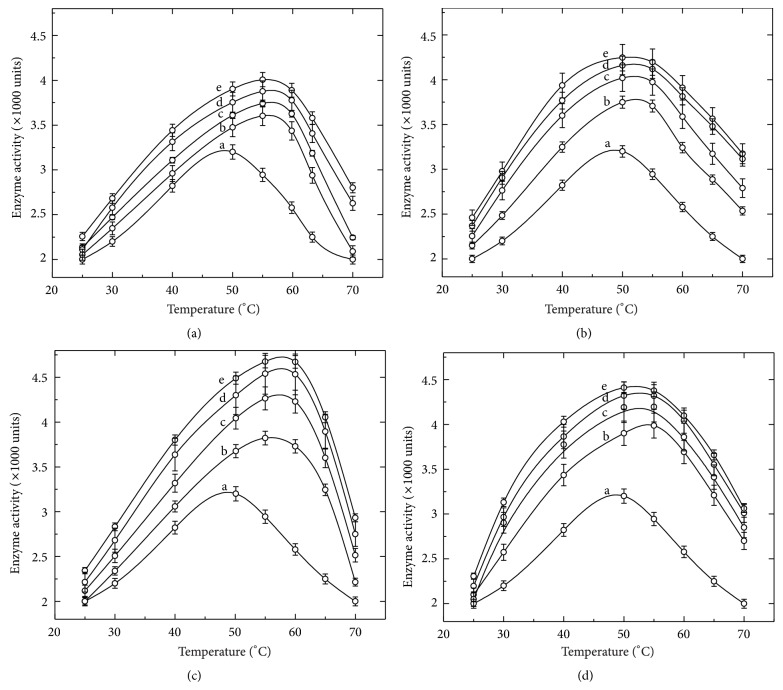
Effect of cosolvents on activity and optimum temperature of *α*-amylase. Figures 2(a), 2(b), 2(c), and 2(d) represent the activity profiles of the enzyme in the presence of glycerol, sorbitol, sucrose, and trehalose, respectively. The activity of the enzyme was measured at different temperatures and curves represented as the enzyme (a) in buffer only, (b) in 10%, (c) in 20%, (d), in 30%, and (e) in 40% (w/v) of respective cosolvents.

Although the increase in thermal resistance of the enzyme appeared attractive, it was not surprising as the enhancement in the thermal stability of various enzymes and proteins in the presence of cosolvents is well documented previously [19–21]. However, the simultaneous observation of the cosolvent-mediated enhancement in the catalytic activity of the enzyme was unexpected. Mere presence of cosolvents significantly improved the catalytic efficiency of the enzyme. The cosolvent-induced enhancement in activity was further noticeable when the activity was measured at higher temperatures, as shown in Figure 2. Moreover, a shift in the temperature for optimum enzyme activity was also observed, at least, in the presence of sucrose and glycerol. As shown in Figure 2, the enzyme in buffer has optimum activity around 50°C. In the presence of cosolvents the enzyme activity was found to be higher at >50°C. The enhancement in the enzyme activity was found to dependent on the concentration of each cosolvent used, at least in the range of 10–40% (w/v). This finding could be of vital importance in the processes where the enzymatic activity is carried out at higher temperature (e.g., starch saccharification and liquefaction, temperature reaches up to 70–100°C). Further, such enhancement in the enzyme activity was found to be reversible.  Table 1 lists the activity of the enzyme before treatment with cosolvents and after their removal from enzyme solution. After removal of cosolvents the enzyme activity was found to be nearly identical to the enzyme without any treatment. 

### 3.3. Cosolvents Induced Structural Perturbation of *α*-Amylase

**Figure 3:**
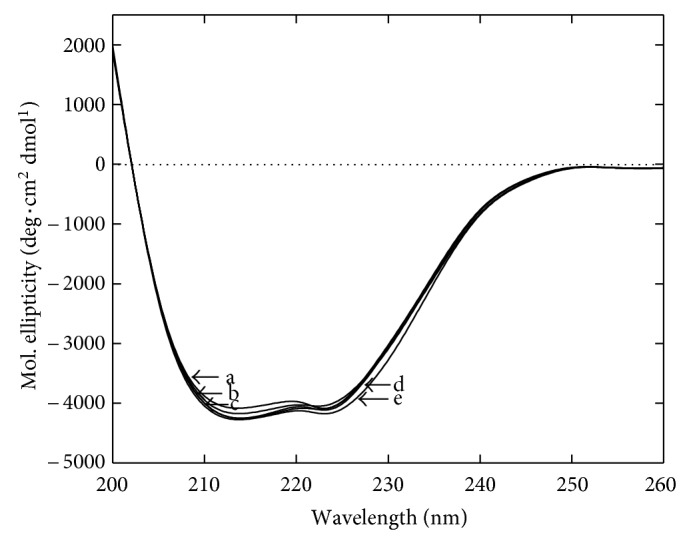
Far-UV CD spectra of *α*-amylase in the presence of cosolvents. Residual molar ellipticity was measured from 200 to 260 nm at 25°C in the absence (a) and presence of 20%  (w/v) glycerol (b), sorbitol (c), sucrose (d) and trehalose (e). Each spectrum represents an accumulation of five independent scans.

**Figure 4:**
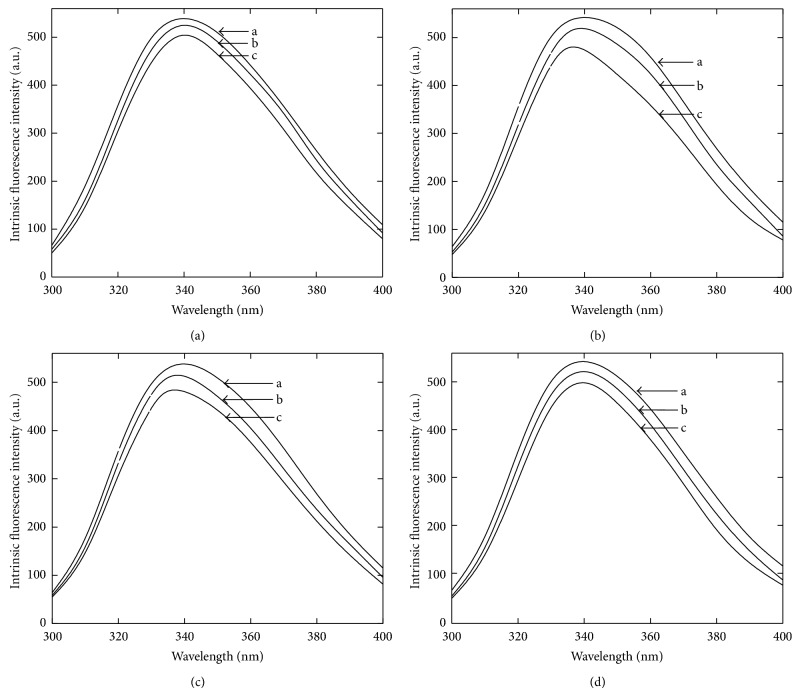
Intrinsic fluorescence of *α*-amylase in the presence of cosolvents. Figures 4(a), 4(b), 4(c), and 4(d) represent the activity profiles of the enzyme in the presence of glycerol, sorbitol, sucrose, and trehalose, respectively. The curves are represented as (a) the enzyme in buffer, (b) in 20%, and (c) in 40% of respective cosolvents.

**Figure 5:**
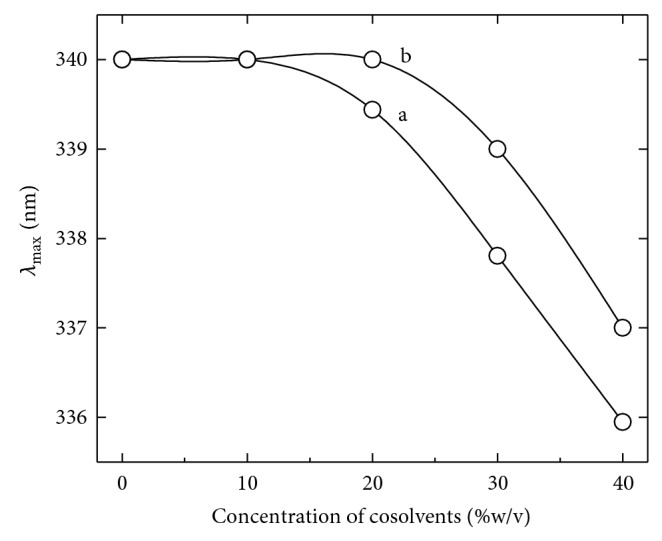
Effect of cosolvents on *λ*_max_ of *α*-amylase. The curves represent the changes in emission maxima of the enzyme in the presence of different concentrations of sucrose (a) and sorbitol (b).

After having observed the effect of cosolvents on the enzyme function and stability, a curiosity developed to investigate the biophysical properties of the enzyme in the presence of cosolvents. The structural analyses of the enzyme in the presence of cosolvents were performed by using far UV-CD and intrinsic fluorescence measurement at ambient temperature. The data from far UV-CD measurement indicate a minor change in the ellipticity in the presence of 20% (w/v), as shown in Figure 3. A slight negative increase in the peaks at 222 nm and 216 nm indicates a minor change in *α*-helical and *β*-sheet components. The intrinsic fluorescence intensity of the enzyme was found to be slightly decreased in the presence of all the cosolvents as shown in Figure 4. In addition, an small shift in the emission maxima (*λ*_max_) was also observed at least in the presence of sucrose and sorbitol. The *λ*_max_ is an excellent parameter to monitor the polarity of tryptophan microenvironment in enzyme molecule and it is sensitive to change in protein conformation [22]. The *λ*_max_ of *α*-amylase in buffer was found to be 340 nm and it was found to be 336 nm and 337 nm in the presence of 40% (w/v) sucrose and sorbitol, respectively (Figure 5). A reduction in intrinsic fluorescence intensity and shifting of *λ*_max_ towards lower wavelength indicate that the aromatic residues are being relocated in more hydrophobic environment [23]. Together with far-UV-CD and fluorescence data it is clear that the presence of cosolvents makes some changes in the enzyme structure which may be accountable for tremendous effect on activity and thermal stability.

## 4. Discussion

It is known that the cosolvents do not interact covalently with protein molecule but confer its stabilizing effect essentially by altering the physicochemical properties of solution, particularly in the vicinity of proteins. In principle, the effect of cosolvents on enzyme activity depends on cosolvent induced alteration in chemical potential of the enzyme. It is generally noticed that the preferentially excluded cosolvents increase the chemical potential of protein. Such partitioning of protein and cosolvents in a solution is likely to alter the equilibrium of enzyme-catalyzed reaction [24]. Such perturbing effect of cosolvents may include a number of processes such as binding of substrates, reversible structural changes, enzyme stability, change in hydration at protein surface, and so forth [25]. It is observed that the presence of cosolvents does not bring about any significant change in the secondary structure of the enzyme. Previous reports indicate that at room temperature, the enzyme is relatively hydrated and the cosolvents were preferentially excluded from the protein-water interface [20, 23]. This means that the surface of enzyme has more concentrated water molecules compared to the bulk solution and interaction of enzyme molecules with cosolvents is less preferred compared to water. The preferential interaction properties are likely to alter with change in temperatures and therefore may influence the stability and function of the enzyme. Although such partitioning does not change the structure of the enzyme to a great extent (as evident from fluorescence and CD measurement), the preferential hydration may induce a tendency to repel the hydrophobic residues that are relatively present on protein surface, in the core regions to avoid thermodynamically unfavorable interactions with water [19]. This may add to increase the hydrophobic interactions, which subsequently enhance the structural stability of proteins. Such a minor structural readjustments may also persuade their effect on the electrostatics of the catalytic sites and render the enzyme to acquire more active state. From the above-described results it appears that the thermal stability of *α*-amylase is significantly improved in the presence of cosolvents. The alteration in the solvent composition due to presence of cosolvents has a positive outcome on the nature of interacting forces responsible for structural reorganization, which provide stability and catalytic efficiency to the enzyme. This finding reveals that an enzyme under macromolecular crowding conditions continued to be catalytically more robust and stable compared to the one in isolation, despite the fact that they possess nearly identical structures.

## Figures and Tables

**Table 1:** Reversibility of cosolvent's induced enhancement of *α*-amylase activity. The enzyme activity was measured after treatment with cosolvents and removal of cosolvents from the protein solution.

Enzyme activity (×1000 unit)			
Cosolvents	Without any treatment	After treatment with cosolvents (40%, w/v)	After removal of cosolvents
Buffer only	2.6 ± 0.15		
Glycerol		3.6 ± 0.1	2.6 ± 0.25
Sorbitol		3.7 ± 0.3	2.8 ± 0.15
Sucrose		3.8 ± 0.25	2.4 ± 0.1
Trehalose		4.1 ± 0.3	2.7 ± 0.2

## References

[1] Wang W. (2005). Protein aggregation and its inhibition in biopharmaceutics. *International Journal of Pharmaceutics*.

[2] Chang L. L., Shepherd D., Sun J. (2005). Mechanism of protein stabilization by sugars during freeze-drying and storage: native structure preservation, specific interaction, and/or immobilization in a glassy matrix?. *Journal of Pharmaceutical Sciences*.

[3] Ragoonanan V., Aksan A. (2007). Protein stabilization. *Transfusion Medicine and Hemotherapy*.

[4] Fujita Y., Iwasa Y., Noda Y. (1984). Effect of polyhydric alcohols on invertase stabilization. *Bulletin of the Chemical Society of Japan*.

[5] Gekko K. (1982). Calorimetric study on thermal denaturation of lysozyme in polyol-water mixtures. *Journal of Biochemistry*.

[6] Arakawa T., Timasheff S. N. (1982). Preferential interactions of proteins with salts in concentrated solutions. *Biochemistry*.

[7] Timasheff S. N. (1993). The control of protein stability and association by weak interactions with water: how do solvents affect these processes?. *Annual Review of Biophysics and Biomolecular Structure*.

[8] Burg M. B., Ferraris J. D. (2008). Intracellular organic osmolytes: function and regulation. *The Journal of Biological Chemistry*.

[9] Bounedjah O., Hamon L., Savarin P., Desforges B., Curmi, Pastre D. (2012). Macromolecular crowding regulates assembly of mRNA stress granules after osmotic stress. *The Journal of Biological Chemistry*.

[10] Hamon L., Savarin P., Curmi P. A., Pastre D. (2011). Rapid assembly and collective behavior of microtubule bundles in the presence of polyamines. *Biophysical Journal*.

[11] Zimmerman S. B., Trach S. O. (1991). Estimation of macromolecule concentrations and excluded volume effects for the cytoplasm of *Escherichia coli*. *Journal of Molecular Biology*.

[12] Farber G. K., Petsko G. A. (1990). The evolution of *α*/*β* barrel enzymes. *Trends in Biochemical Sciences*.

[13] Janecek S. (1997). Alpha-amylase family: molecular biology and evolution. *Progress in Biophysics and Molecular Biology*.

[14] MacGregor E. A. (1988). *α*-Amylase structure and activity. *Journal of Protein Chemistry*.

[15] Holm L., Koivula A. K., Lehtovaara P. M., Hemminki A., Knowles J. K. C. (1990). Random mutagenesis used to probe the structure and function of *Bacillus stearothermophilus* alpha-amylase. *Protein Engineering*.

[16] Lowry O. H., Rosenbrough N. J., Farr A. L., Randall R. J. (1951). Protein measurement with the Folin phenol reagent. *The Journal of Biological Chemistry*.

[17] Bernfeld P. (1955). *α*- and *β*-Amylases. *Methods in Enzymology*.

[18] Declerck N., Machius M., Joyet P., Wiegand G., Huber R., Gaillardin C. (2003). Hyperthermostabilization of *Bacillus licheniformisα*-amylase and modulation of its stability over a 50°C temperature range. *Protein Engineering*.

[19] Lee J. C., Timasheff S. N. (1981). The stabilization of proteins by sucrose. *The Journal of Biological Chemistry*.

[20] Rajendran S., Radha C., Prakash V. (1995). Mechanism of solvent-induced thermal stabilization of *α*-amylase from *Bacillus amyloliquefaciens*. *International Journal of Peptide and Protein Research*.

[21] Timasheff S. N. (1998). Control of protein stability and reactions by weakly interacting cosolvents: the simplicity of the complicated. *Advances in Protein Chemistry*.

[22] Santucci R., Polizio F., Desideri A. (1999). Formation of a molten-globule-like state of cytochrome c induced by high concentrations of glycerol. *Biochimie*.

[23] Yadav J. K., Prakash V. (2009). Thermal stability of alpha-amylase in aqueous cosolvent systems. *Journal of Biosciences*.

[24] Timasheff S. N. (2002). Protein-solvent preferential interactions, protein hydration, and the modulation of biochemical reactions by solvent components. *Proceedings of the National Academy of Sciences of the United States of America*.

[25] Timasheff S. N. (2002). Protein hydration, thermodynamic binding, and preferential hydration. *Biochemistry*.

